# Supplementary material for: Automatic segmentation of the great arteries for computational hemodynamic assessment
Source: J Cardiovasc Magn Reson. 2022 Nov 7;24:57. doi: 10.1186/s12968-022-00891-z (PMC9639271; doi:10.1186/s12968-022-00891-z)
Supplement: Supplementary file 1 — Additional file 1. Supplementary Materials A. [file 12968_2022_891_MOESM1_ESM.docx]

# Supplementary Materials A

## Sensitivity analysis laminar vs turbulent

It was found that an element count of ~300,000 was well-suited to capture the flow details in both the aorta and the PA, and for yielding a stable solution. Iterations of 300 and 750 were sufficient for the aorta and PA respectively, in order to reach convergence and accurate results. The error (MAPE) for the aorta with 315,766 elements vs the highest resolution aorta with 1,102,454 elements was 2.02% in pressure, and 3.4% in velocity. The error (MAPE) for the PA with 398,947 elements vs the highest resolution PA with 1,116,831 elements was 3.23% in pressure, and 2.15% in velocity. Lastly, the Reynold’s number was computed on both test cases, and resulted in 3221.8 and 4250.5 for the aorta and pulmonary artery respectively. The same simulations were carried out with a k-omega turbulence model (with default parameters), which showed no difference when compared to the laminar model.


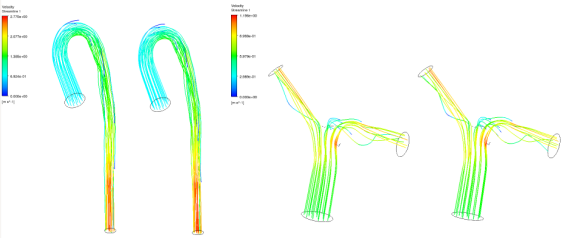


Figure 1: Difference velocity fields in Ao and PA when applying a k-omega turbulence model(left = laminar, right = turbulent).
