# Supplementary material for: Automatic segmentation of the great arteries for computational hemodynamic assessment
Source: J Cardiovasc Magn Reson. 2022 Nov 7;24:57. doi: 10.1186/s12968-022-00891-z (PMC9639271; doi:10.1186/s12968-022-00891-z)
Supplement: Supplementary file 2 — Additional file 2. Supplementary Materials B. [file 12968_2022_891_MOESM2_ESM.docx]

# Supplementary Materials B

## Hyperparameter Optimization

Training, including hyperparameter optimization, took ~24 h, during which a total of 124 hyperparameter configurations were sampled by the Hyperband iterations. The top 10 performing configurations in terms of mean validation Dice score are reported in Table 1 and Figure 1.

Table 1 Top 10 hyperparameter configurations.

The table shows the values for each of the hyperparameters in the search space and the mean validation Dice score.

| # | Scales | Layers per block | Initial filters | Learning rate | Batch size | Loss function | Dice |
| --- | --- | --- | --- | --- | --- | --- | --- |
| 1 | 3 | 2 | 64 | 3.46 ∙ 10^-4^ | 2 | Focal Tversky | 0.946 |
| 2 | 3 | 4 | 32 | 1.99 ∙ 10^-4^ | 2 | Tversky | 0.944 |
| 3 | 4 | 3 | 32 | 3.40 ∙ 10^-4^ | 4 | Dice | 0.944 |
| 4 | 4 | 3 | 32 | 1.37 ∙ 10^-4^ | 4 | IoU | 0.943 |
| 5 | 3 | 3 | 64 | 6.77 ∙ 10^-4^ | 4 | IoU | 0.939 |
| 6 | 3 | 3 | 64 | 3.27 ∙ 10^-4^ | 4 | Dice | 0.938 |
| 7 | 4 | 3 | 32 | 1.81 ∙ 10^-4^ | 2 | Dice | 0.938 |
| 8 | 3 | 4 | 32 | 1.83 ∙ 10^-3^ | 2 | Focal Tversky | 0.937 |
| 9 | 2 | 3 | 32 | 1.24 ∙ 10^-3^ | 4 | Jaccard | 0.935 |
| 10 | 3 | 2 | 32 | 4.68 ∙ 10^-3^ | 4 | Dice | 0.934 |


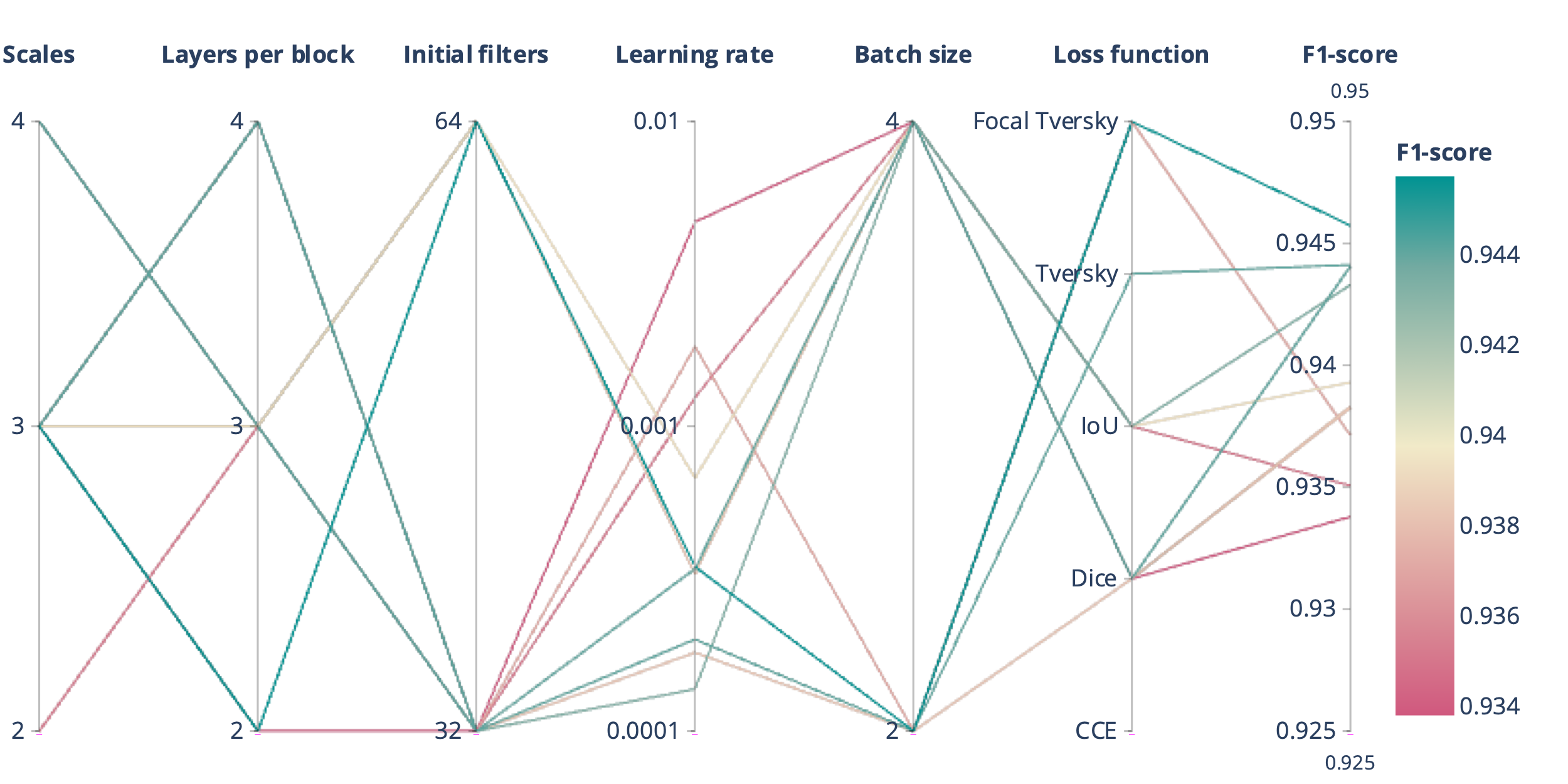


Figure 1 Parallel coordinates view of the top 10 hyperparameter configurations.

Each hyperparameter as well as the mean validation Dice score is shown on its own axis. Each coloured line represents a hyperparameter combination, with vertices at the corresponding values on the parallel axes.

The best performing configuration was as follows: scales = 3, layers per block = 2, initial filters = 64, learning rate = 3.46 ∙ 10^-4^, batch size = 2, and loss function = focal Tversky. This model was selected and used in all further experiments.

The top 8 performing configurations have 3 or 4 scales, which suggests that models with 2 scales may have too limited representational power or deep layer receptive fields. Models with at least 3 layers per block also tended to perform better, with the notable exception of the best performing model, which had 2. A relatively small learning rate also seems to be advantageous, with 6 of the top 7 configurations below 0.0005. Confusion-based losses seem to outperform cross-entropy, which does not appear on any of the top 10 configurations, but none of those appears to consistently outperform the rest. Finally, although our best model had 64 initial filters, the abundance of top performing models with 32 filters suggests it might be possible to find a 32-filter configuration with little or no performance penalty, which would result in a 2x reduction in the number of parameters and computational cost.
