# Supplementary material for: Automatic segmentation of the great arteries for computational hemodynamic assessment
Source: J Cardiovasc Magn Reson. 2022 Nov 7;24:57. doi: 10.1186/s12968-022-00891-z (PMC9639271; doi:10.1186/s12968-022-00891-z)
Supplement: Supplementary file 3 — Additional file 3. Supplementary Materials C. [file 12968_2022_891_MOESM3_ESM.docx]

# Supplementary Materials C

## Difference between manual clipping and equal clipping

In all cases, the equally clipped data produces better agreement between the ML and GT CFD simulations. The median pressure and velocity error in aortas is reduced through equal clipping by 2.1 and 1.0 percentage points, respectively. The median pressure and velocity error in PAs is reduced through equal clipping by 4.2 and 2.7 percentage points, respectively. The magnitude of outliers is also reduced through equal clipping.


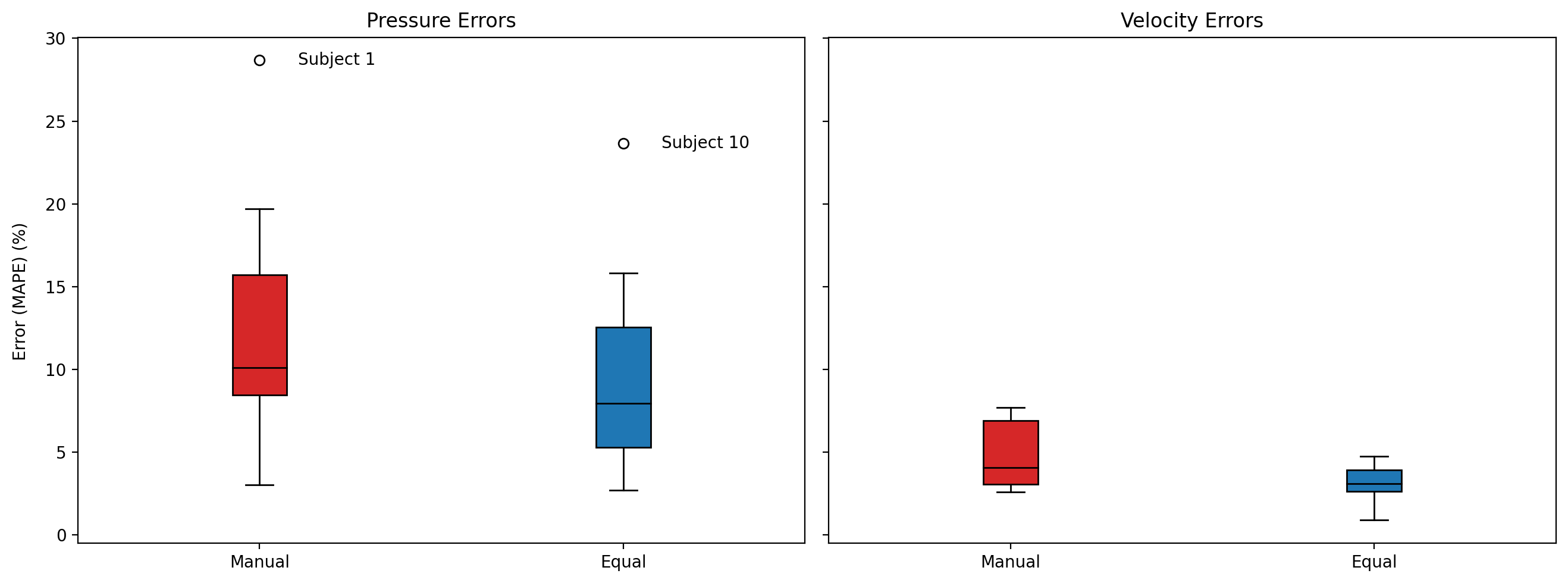
Figure 1: Difference between manually and equally clipped aortas


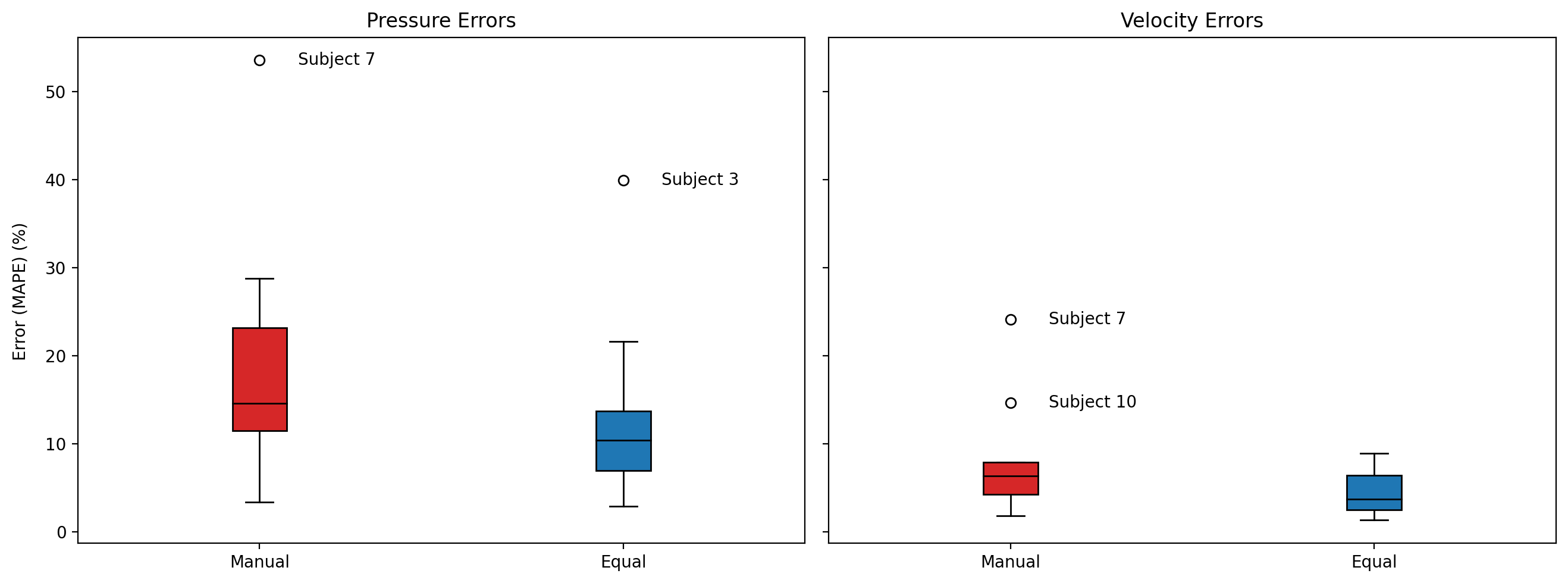
Figure 2: Differences between manually clipped and equally clipped PAs
